# Participation of Hepcidins in the Inflammatory Response Triggered by λ-Carrageenin in Gilthead Seabream (*Sparus aurata*)

**DOI:** 10.1007/s10126-024-10293-0

**Published:** 2024-02-14

**Authors:** Jose Carlos Campos-Sánchez, Jhon A. Serna-Duque, Carmen Alburquerque, Francisco A. Guardiola, María Ángeles Esteban

**Affiliations:** Immunobiology for Aquaculture Group, Department of Cell Biology and Histology, Faculty of Biology, Campus Regional de Excelencia Internacional “Campus Mare Nostrum”, University of Murcia, 30100 Murcia, Spain

**Keywords:** Hepcidins, Antimicrobial peptides, Inflammation, Iron metabolism, Carrageenin, Seabream (*Sparus aurata*)

## Abstract

The role of hepcidins, antimicrobial peptides involved in iron metabolism, immunity, and inflammation, is studied. First, gilthead seabream (*Sparus aurata* L.) head-kidney leucocytes (HKLs) were incubated with λ-carrageenin to study the expression of hepcidin and iron metabolism-related genes. While the expression of most of the genes studied was upregulated, the expression of ferroportin gene (*slc40a*) was downregulated. In the second part of the study, seabream specimens were injected intramuscularly with λ-carrageenin or buffer (control). The expression of the same genes was evaluated in the head kidney, liver, and skin at different time points after injection. The expression of Hamp1m, ferritin b, and ferroportin genes (*hamp1*, *fthb*, and *slc40a*) was upregulated in the head kidney of fish from the λ-carrageenin-injected group, while the expression of Hamp2C and Hamp2E genes (*hamp2.3* and *hamp2.7*) was downregulated. In the liver, the expression of *hamp1*, ferritin a (*ftha*), *slc40a*, Hamp2J, and Hamp2D (*hamp2.5/6*) genes was downregulated in the λ-carrageenin-injected group. In the skin, the expression of *hamp1* and (Hamp2A Hamp2C) *hamp2.1/3/4* genes was upregulated in the λ-carrageenin-injected group. A bioinformatic analysis was performed to predict the presence of transcription factor binding sites in the promoter region of hepcidins. The primary sequence of hepcidin was conserved among the different mature peptides, although changes in specific amino acid residues were identified. These changes affected the charge, hydrophobicity, and probability of hepcidins being antimicrobial peptides. This study sheds light on the poorly understood roles of hepcidins in fish. The results provide insight into the regulatory mechanisms of inflammation in fish and could contribute to the development of new strategies for treat inflammation in farm animals.

**Graphical Abstract:**

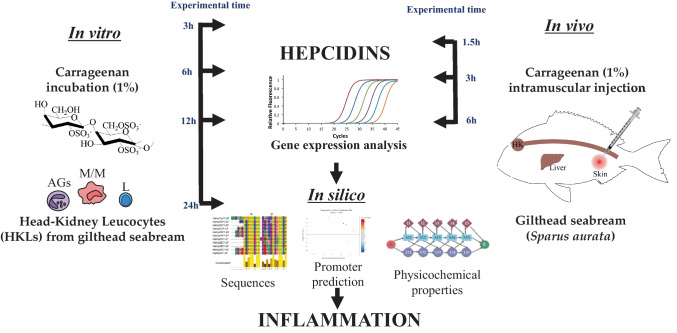

## Introduction

Inflammation is a biochemical sequence of highly coordinated events triggered by the host innate immune system in order to protect the organism against any stimuli such as microbes and irritants and injuries, (Chen et al. 2018). Depending on the cause, a variety of preformed inflammatory mediators are released by tissue-resident immune and non-immune cells. These mediators include cytokines, chemokines, adhesion molecules, proteolytic proteins, antimicrobial peptides, histamines, prostaglandins, leukotrienes, neuropeptides, and neurotransmitters. These molecules increase blood flow, vasodilation, and vascular permeability, allowing for the production of immunoglobulins, acute-phase reactants, coagulation factors, complement activation, and the recruitment of leukocytes from the blood capillaries to the surrounding tissues and ultimately to the site of inflammation (Calder et al. 2013; Kolaczkowska and Kubes 2013). However, once the initial damage has been eliminated, regulatory mechanisms of homeostasis are activated to terminate acute inflammation and promote healing (Campos-Sánchez and Esteban 2021).

Although pathogens and trauma can trigger an acute inflammatory response within minutes or hours, the ineffective removal of foreign substances or the persistence of the active inflammatory mechanism for an extended period of time (weeks, months, or years) can exacerbate the process and render it chronic (Libby 2007; Muller 2013). Moreover, chronic inflammation is considered a hallmark of several autoimmune, cancerous, and metabolic diseases (Libby 2007; Muller 2013). In mammals, inflammation is typified by a variety of characterized specific symptoms, including heatsensation, redness, swelling, pain, and functional disorders (Nathan 2002; Chen and Nuñez 2010). However, the inflammation sequelae in fish species have not been thoroughly studied.

In recent decades, increasing global fish consumption and high market demands have made aquaculture one of the fastest-growing food production sectors worldwide (FAO 2020). However, the intensive aquaculture practices lead to the occurrence of frequent skin lesions and inflammations in farmed fish, as well as disease outbreaks (mostly caused by bacteria or viruses), which are associated with high mortalities and production losses (Chen and Nuñez 2010; Balcázar et al. 2006; Esteban 2012). Traditionally, fish farmers have relied on the use of antibiotics to prevent diseases in aquaculture despite their negative effects (Cabello 2006). In this sense, the development of bioinformatics techniques has allowed the identification and characterization of related inflammatory markers to find therapeutic agents in aquaculture. Among them, peptides have been mostly designated as host defense peptides (HDPs) due to their ability to exhibit broad-spectrum antimicrobial activity against bacteria, viruses, parasites, and fungi (Mills et al. 2015; Gupta et al. 2018). AMPs can also develop immunomodulatory and pro- or anti-inflammatory actions through their insertion into biological membranes (Mills et al. 2015; Gupta et al. 2018). AMPs are a broad group of small cationic and hydrophobic molecules (18–46 amino acids) widely distributed from invertebrates to mammals (Katzenback 2015). As previously commented, AMPs are mediators induced during the innate immune response against pathogens, although they can also be produced at the constitutive level (Katzenback 2015). In fish, several AMPs have been identified and classified according to their phylogenetic conservation into five main families: (i) hepcidins, (ii) β-defensins, (iii) cathelicidins, (iv) histone-derived peptides, and the fish-specific (v) piscidins (Cuesta et al. 2008; Katzenback 2015).

Hepcidin is a small cysteine-rich peptide (composed of six to eight conserved cysteines), which belongs to the group of highly disulfide-bonded β-sheet peptides, and is mainly produced by hepatocytes, and secreted into serum, playing an important role in iron metabolism and innate immunity (Krause et al. 2000). The mechanism involves the reduction of iron to Fe^2+^ to enter the hepatocyte (Schmidt 2015). It can then be sequestered in the form of ferritin or transferred across the basolateral membrane by ferroportin, where it is oxidized to Fe^3+^ and loaded into transferrin, a protein that transports and distributes iron to the tissues that need it (Schmidt 2015). Therefore, three important proteins including ferroportin (iron exporter), transferrin (serum iron transporter), and ferritin (iron store) are involved in the regulation of mammalian iron balance, which can serve as iron biomarkers (Ueda and Takasawa 2018). Following the iron overload, caused by infection or inflammation, hepcidin is induced to posttranslationally inhibit iron-exporting ferroportin, resulting in its internalization and degradation (Nemeth and Ganz 2006). This fact leads to a decrease in iron release to plasma from hepatocytes, macrophages, and enterocytes (Nemeth and Ganz 2006; Serna-Duque et al. 2022a). As a result, circulating iron is limited, being unavailable for bacterial growth. This process must be tightly regulated, as long-term iron retention may also affect intestinal iron absorption and iron availability for erythropoiesis, resulting in inflammatory anemia (Nemeth and Ganz 2006; Viatte and Vaulont 2009; Ganz 2011). In this regard, hepcidin is inhibited by iron deficiency and hypoxia (Nemeth and Ganz 2006; Viatte and Vaulont 2009; Ganz 2011). However, this regulatory mechanism of hepcidin has not yet been sufficiently demonstrated in fish. Specifically, it has only been studied in a few fish species such as *Danio rerio* (Jiang et al. 2017), *Salmo trutta* (Huang et al. 2019), *Oncorhynchus mykiss* (Álvarez et al. 2014), *Dicentrarchus labrax* (Rodrigues et al. 2006), or *Sparus aurata* (Cuesta et al. 2008). Recently, the expression of hepcidins has also been studied during the larval development of zebrafish (Caccia et al. 2017) and in some adult fish species [*Ictalurus punctatus* (Bao et al. 2006), *Oreochromis mossambicus* (Huang et al. 2007) *Cyprinus carpio* (Yang et al. 2014) *Sebastes schlegelii* (Ma et al. 2020), and *Acrossocheilus fasciatus* (Zhu et al. 2023)].

To extend our knowledge on the mechanism of action of hepcidin in fish inflammation, we used carrageenin as an induction model of acute sterile inflammation. Carrageenin is a high molecular weight sulfated mucopolysaccharide obtained from the cell walls of red algae (family Rhodophyceae) whose structure is composed of α (1, 3)—β (1, 4)—galactans with one (κ-), two (ι-), or three (λ-) sulfates per disaccharide unit (Necas and Bartosikova 2013). This degree of sulfation play a key role in triggering inflammation in rodents, being λ-carrageenin the main form used for this purpose (Winter et al. 1962; Levy 1969; Fujiki et al. 1997; Morris 2003; Bhattacharyyaa et al. 2010; Necas and Bartosikova 2013; Sokolova et al. 2014). Carrageenin also triggers inflammation in several fish species [carp (*Cyprinus carpio*) (Fujiki et al. 1997), Nile tilapia (*Oreochromis niloticus*) (Matushima and Mariano 1996), zebrafish (*D. rerio*) (Huang et al. 2014; Ribas et al. 2016; Belo et al. 2021), and gilthead seabream (*S. aurata*)] (Campos-Sánchez et al. 2021b, c, d). Recently, we have optimized an in vivo carrageenin-induced acute inflammation model for gilthead seabream (Campos-Sánchez et al. 2021a; 2022a). In addition, the use of non-invasive techniques such as real-time ultrasound or micro-CT has also provided high-quality images of this complex process (Campos-Sánchez et al. 2022b). Taking into account our previous results, the present study aimed to evaluate the role of hepcidins in various tissues of seabream after triggering acute experimental inflammation by intramuscular injection of λ-carrageenin. To our knowledge, this is the first study using bioinformatics techniques to characterize the inflammatory mechanism in fish.

## Material and Methods

### Animals

Gilthead seabream (*S. aurata* L.) specimens were obtained from a local farm (Mazarrón, Spain) and were kept in re-circulating seawater aquaria (450 L) in the Marine Fish Facilities at the University of Murcia (Spain) during a quarantine period of 1 month. The water temperature was maintained at 20 ± 2 °C with a flow rate of 900 L h^−1^, 28‰ salinity, a photoperiod of 12 h light to 12 h dark, and with continuous aeration. Water ammonium and nitrite levels were monitored in the tanks with specific kits (Seachem) and were maintained below the limits for the species (0.1 mg L^−1^ and 0.2 mg L^−1^, respectively). Fish were fed with a commercial diet (Skretting, Spain) at a rate of 2% body weight day^−1^ and were kept 24 h without feeding before the trial. All experimental protocols were approved by the Ethical Committee of the University of Murcia (permit number A13160416).

### In Vitro Experiment

#### Head-Kidney Leucocytes Isolation and Incubation with Carrageenin

Six fish (weight 284.53 g ± 17.21 g, and length 26.02 cm ± 0.39 cm) were randomly selected from a pool of 50 fish and anesthetized with clove oil (20 mg L^−1^, Guinama^®^), and bleed from the caudal vein. Head kidney (HK) were dissected out by ventral incision, cut into small fragments, and transferred to 12 mL of sRPMI [RPMI culture medium (Gibco) supplemented with 0.35% sodium chloride (to adjust the medium’s osmolarity to gilthead seabream plasma osmolarity of 353.33 mOs), 3% fetal calf serum (FCS, Gibco), 100 i.u. mL^−1^ penicillin (Flow) and 100 mg mL^−1^ streptomycin (Flow)] (Esteban et al. 1998). Suspensions of head-kidney leucocytes (HKLs) were obtained by forcing the organ fragments through a nylon mesh (mesh size 100 µm). The HKLs were washed twice by centrifugation (400 × *g* 10 min), and adjusted to 2 × 10^7^ cells mL^−1^ in sRPMI. Cell viability was higher than 98%, as determined by the trypan blue exclusion test using TC20 automated cell counter (Bio-Rad).

λ-carrageenin (Sigma) was diluted in sterile phosphate-salt buffer (PBS; 11.9 mM phosphate, 137 mM NaCl, and 2.7 mM KCl, pH 7.4) (Fisher Bioreagents), and a stock solution of 10 mg mL^−1^ was prepared and resuspended in sRPMI. Prior to assays, the osmolarity of these solutions was measured in an osmometer (Wescor) to avoid effects due to this parameter. For each fish, 500 µL of the isolated HKLs were dispensed in 1.5 mL Eppendorf with 500 µL of λ-carrageenin dilutions to make final concentrations of 0 µg mL^−1^ (PBS diluted in sRPMI; control) and 1000 µg mL^−1^, and then incubated for 3, 6, 12, or 24 h, at 25 °C and 5% CO_2_.

#### Gene Expression by Real-Time PCR

The sequences of the selected genes were obtained from a gilthead seabream database (Pareek et al. 2011). Open reading frames (ORFs) were located using the ExPASy translation software (SIB Bioinformatics Resource Portal), and further checking was performed using NCBI BLAST sequence alignment analysis (NIH). Primers were designed using the OligoPerfect™ tool (Thermo Fisher), according to the following criteria: (i) each oligonucleotide was composed of 20 nucleotides; (ii) amplicon size was between 100 and 120 nucleotides; (iii) with a % guanidine-cytosine (GC) between 55 and 60%; (iv) a melting temperature as close as possible to 60 °C; and (v) the selection of primers that self-inhibit by forming hairpins were avoided as much as possible, so as not to hinder the amplification reaction. The primers used are presented in Table 1.

**Table 1:** Primers used for real-time qPCR

**Peptide**	**Gene name**	**GenBank number**	**Primer sequences (5′ → 3′)**
Hamp1m	*hamp1*	115566792	F: AAGCGTCAGAGCCACATCTC R: AGTCAATGCGTCGGAGAAGG
Hamp2A	*hamp2.1*	115567002	F: ATGTGGTGTCTGCTGCACATT R: GCAGCATGACCAAATCCAGAGAT
Hamp2B	*hamp2.2*	115567007	F: CCTGACACGACTGGATGTAATGT R: GCATGACCAAATCCAGGAACATCC
Hamp2C	*hamp2.3*	BK059173	F: AGCAGCTTTCCAAATTTCCTTAGT R: TTAGGATGATACATCAGTTAGCACA
Hamp2C	*hamp2.4*	BK059174	F: CAGCCTGGGGTTCACACAAC R: ACTGATCACACATGAAGGAGGATG
Hamp2J	*hamp2.5*	115567006	F: AGATGGGGTATGGCAACAGG R: AATGCAATTTGGAGAACTGTTTATG
Hamp2D	*hamp2.6*	115567004	F: TGCTGTCCCATTCACTAAGGT R: CAAAACTTACACCTCCTGCG
Hamp2E	*hamp2.7*	BK059175	F: GGGATTCACACAACAACCACTG R: GAAGATTCTTGAGGATGATACAGTCAC
Hamp2F	*hamp2.8*	115566998	F: AGCCTGGGATTCACACAAC R: GATTTGACACTTTCAGTTAAAAAGGTT
Hamp2E	*hamp2.9*	BK059176	F: CGGTTGCTGTCCTAACATGA R: GACCAAATCCAGATATTACATCCTC
Hamp2G	*hamp2.10*	115567005	F: CCGCTGGCTGTAAGTTTTGC R: TTGTGTGAATCCCAGGCTGC
Hamp2E	*hamp2.11*	115567000	F: CTGGGATTCACACAACAACCA R: TGCGACTGTATCACCTACAC
Hamp2H	*hamp2.12*	115567003	F: CCGCTGGCTGTAAGTTTTGT R: TTGTGTGAATTCCAGACTGC
Hamp2E	*hamp2.13*	115566999	F: TGGGATTCACACAACAACAACTT GTAGCGTGTGTTGGTGATACAGTC
Hamp2I	*hamp2.14*	115567001	F: AGCCCTGCTGACTGTGAGTT R: ACAGCCACAAAAGGAGTGCAA
Ferritin a	*fth1a*	115580735	F: CCTCAGAATGGCATGGCAGA R: AGCCGGTATCATGCAGATGG
Ferritin b	*fth1b*	115586471	F: CAAACACACCATGGCCGAAG R: TGCAGTACATGATGGGGAGC
Ferroportin	*slc40a1*	115587845 115587844	F: TAAAGTGGCCCAGACCTCGC R: GGATGTAGCAGGTCGTCAGAAT
Transferrin	*tf*	115572354	F: CAGGACCAGCAGACCAAGTT R: TGGTGGAGTCCTTGAAGAGG
Ribosomal protein S18	*rps18*	AM490061	F: CGAAAGCATTTGCCAAGAAT R: AGTTGGCACCGTTTATGGTC
Elongation factor-1 alpha	*ef1a*	AF184170	F: TGTCATCAAGGCTGTTGAGC R: GCACACTTCTTGTTGCTGGA

After 3, 6, 12, or 24 h incubation of HKLs with both concentrations of λ-carrageenin (0 -control- and 1000 μg mL^−1^), Eppendorfs were centrifuged (400 × *g*, 10 min, 22 °C), and the obtained pellet containing the cells was stored at −80 °C for gene expression analysis. Total RNA was extracted from HKLs with the PureLink^®^ RNA Mini Kit (Life Technologies) according to the manufacturer’s instructions, and quantification and purification were assessed using the Nanodrop^®^ spectrophotometer; 260:280 ratios were 1.8–2.0. The RNA was then treated with DNase I (Promega) to remove genomic DNA contamination.

Complementary DNA (cDNA) was synthesized from 1 µg of total RNA using the SuperScript IV reverse transcriptase (Life Technologies) with an oligo-dT_18_ primer. In the present study, the expression of selected genes was assessed by real-time qPCR with QuantStudio™ Real-Time PCR System Fast (Life Technologies). Reaction mixtures [containing 5 µL of SYBR Green supermix, 2.5 µL of primers (0.6 µM each), and 2.5 µL of cDNA template] were incubated for 10 min at 95 °C, followed by 40 cycles of 15 s at 95 °C, 1 min at 60 °C, and finally 15 s at 95 °C, 1 min at 60 °C, and 15 s at 95 °C. The gene expression was analyzed using the 2^−ΔCt^ method (Livak and Schmittgen 2001), which was performed as described elsewhere (Cordero et al. 2015). The specificity of the reactions was analyzed using samples without cDNA as negative controls. For each mRNA, gene expression was normalized with the geometric mean of ribosomal protein (*s18*) and elongation factor 1-alpha (*ef1a*) RNA content in each sample. Gene names follow the accepted nomenclature for zebrafish (http://zfin.org/). In all cases, each PCR was performed on triplicate samples.

### In Vivo Experiment

Twenty-four seabream specimens (15.84 ± 4.21 g, 10.23 cm ± 0.85 cm) were randomly selected, anesthetized with clove oil (20 mg L^−1^, Guinama^®^), and intramuscularly injected in the left flank, beneath the lateral line at the level of the second dorsal fin. Two groups of fish (with two replicates per group) were established: (i) fish injected with 50 µL of PBS (Fisher Bioreagents) (control group), and (ii) fish injected with 50 µL of λ-carrageenin (1%, Sigma-Aldrich) in PBS. After 1.5-, 3-, and 6-h post-carrageenin injection (p.i.), three fish from each tank (*n* = 6 per group) were sedated as previously described, weighed, measured, and sampled. The HK was obtained as previously described), and samples of liver and skin were collected and stored at − 80 °C till use. Gene expression analysis was developed as previously described.

### In Silico Analysis

#### Sequence Analysis of Hepcidin Proteins

Protein sequences were obtained from the UniProtKB database (https://www.uniprot.org/), using the entry Pfam PF06446 (hepcidin) as a query. Mature hepcidin sequences were obtained after analysis of the signal peptide cleavage sites with SignalP 6.0 and mature peptides after furin action with ProP 1.0 (https://services.healthtech.dtu.dk/) (Serna-Duque et al. 2022b). Gilthead seabream hepcidin protein sequences were compared by multiple alignments (Clustal Omega, EMBL-EBI) and visualized by Jalview 2.11. Calculations and estimations on the physicochemical properties of the peptides were performed by APD3 calculator (Antimicrobial Peptide Database), and antimicrobial activity was predicted in CAMPR3 using the algorithm Random Forest.

#### Enrichment of Hepcidin Promoter Prediction

The core promotors and cis-transcription factor binding sites (TFBS) in the 5′-flanking region upstream of seabream hepcidin genes (−1000 bp from the start codon; Table 3) were studied. TATA boxes and transcription start sites (TSS) were performed by *TSSG (Softberry)* and *BDGP Neural Network Promoter Prediction*. TFBS were predicted for all hepcidin genes by Vertebrates JASPAR 2020 Core matrix with deficit cut-off of 0.05 in CiiDER (https://doi.org/10.1371/journal.pone.0215495). Enrichment analysis was performed from the above result with 21 promoter sequences of inflammation-related genes (Table 3) as background genes, and 0.05 enrichment coverage *p*-value*.* Finally, it was visualized in site map and enrichment plot using the CiiiDER tool.

### Statistical Analysis

The results were expressed as mean relative expression (fold change with respect to controls) ± standard error of the mean (SEM) or color intensity in the heat maps. Data were analyzed by Student’s *t*-test, and one-way ANOVA (followed by Tukey’s tests) to determine differences between experimental groups and each group with respect to time, respectively. The normality of the data was previously assessed using a Shapiro-Wilk test, and the homogeneity of variance was also verified using the Levene test. Non-normally distributed data were log-transformed to perform parametric tests, and when the data did not meet parametric assumptions, a nonparametric Mann–Whitney *U* test, or a Kruskal–Wallis test followed by Dunn’s multiple comparison test, was used. With respect to computational enrichment of over- and underrepresented hepcidin promoters, TFs were determined by comparing the number of sequences with predicted TFBS to the number of those without, using Fisher’s exact test. All statistical analyses were conducted using the computer package SPSS (25.0 version; SPSS Inc., Chicago, IL, USA) for Windows. The level of significance used was *p* < 0.05 for all statistical tests.

## Results

### In Vitro Experiment

**Fig. 1:**
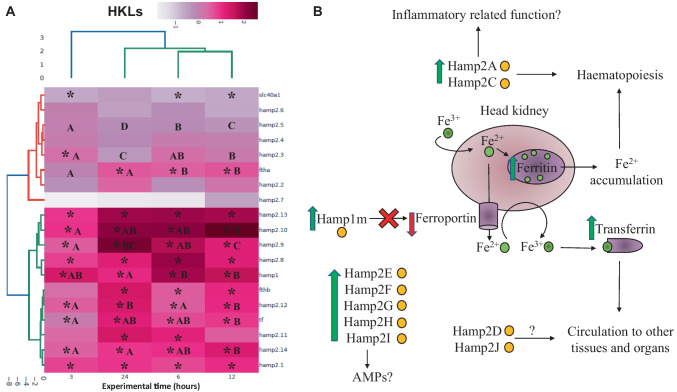
Results of the in vitro experiment. **A** Heat map of the relative expression of hepcidins and iron metabolism-related genes in head-kidney leucocytes (HKLs) of gilthead seabream after incubation with 0 and 1000 µg mL^−1^ of λ-carrageenin for 3, 6, 12, and 24 h. The color scale at the top of the heat map represents the level of gene expression. Data are represented as log10 of fold change expression normalized to the endogenous control *s18* and *ef1a* gene. Asterisks indicate significant differences between control and λ-carrageenin groups (*t*-test; *p* < 0.05), and different letters indicate differences between time points (ANOVA; *p* < 0.05). **B** Proposed schematic model of the role of hepcidins after incubation of gilthead seabream head-kidney leucocytes with λ-carrageenin. The green and red arrows represent the up- and the downregulation of the studied genes, respectively, from the gene expression data found in the in vitro assay

The results showed that the expression of most of the hepcidin genes studied (*hamp1*, *hamp2.1*, *hamp2.8*, *hamp2.9*, *hamp2.10*, *hamp2.12*, *hamp2.13*, and *hamp2.14*) were upregulated in HKLs after incubation with λ-carrageenin at all experimental times tested (Fig. 1A). In addition, the expression of *hamp2.3* and *hamp2.11* genes was significantly (*p* < 0.05) increased in HKLs at 3 h and at 6 and 24 h of incubation with λ-carrageenin, respectively. As for iron-metabolism genes, the expression of *ftha* and *fthb* genes was significantly increased in HKLs after incubation with λ-carrageenin for more than 6 h. Transferrin gene expression increased in all HKL incubated with λ-carrageenin compared to the values recorded in control HKL. In contrast, *slc40a* gene expression decreased in HKLs at 3, 6, and 12 h after incubation with λ-carrageenin. The expression values of *hamp2.2/4/5/6/7* genes showed no significant variations. Taking into account the time factor, the expression of the *hamp1* and *ftha* genes was upregulated at 6 and 12 h of incubation with λ-carrageenin compared to 24 h of incubation. In the case of the *ftha* gene, this positive regulation was also significant with respect to 3 h of incubation. In addition, the expression of *hamp2.10*, *hamp14*, *ftha*, and *tf* was upregulated at 12 h of incubation with λ-carrageenin relative to that at 3 h of incubation. This increase was also significant at 6 h of incubation in the case of *ftha.* Furthermore, the gene expression of *hamp2.9* and *hamp2.14* was upregulated in a time-dependent manner, while the expression of *hamp2.3* and *hamp2.5* genes was downregulated with increasing incubation time.

A schematic model representing the possible effects of λ-carrageenin on HKLs was proposed in Fig. 1B.

### In Vivo Experiment

**Fig. 2:**
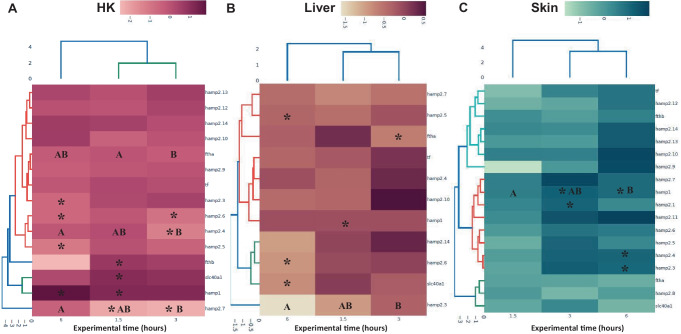
Results of the in vivo experiment. Heat map of the relative expression of hepcidins and genes related to iron metabolism in **A** head kidney, **B** liver, and **C** skin samples of gilthead seabream injected with PBS (control) or λ-carrageenin (1%) and sampled at 1.5, 3, and 6 h post-injection. The color scale at the top of the heat map represents the level of gene expression. Data are represented as log10 of fold change expression normalized to endogenous control *s18* and *ef1a* gene. Asterisks indicate significant differences between control and λ-carrageenin groups (*t*-test; *p* < 0.05), and different letters indicate differences between time points (ANOVA; *p* < 0.05)

The results revealed that *hamp1*, *fthb*, and *slc40a* gene expression was significantly upregulated, while *hamp2.7* gene expression was downregulated, in HK of fish injected with λ-carrageenin and sampled at 1.5 h p.i., compared with the data observed in the control group (Fig. 2A). In the HK of fish injected with λ-carrageenin, at 3 h p.i., the expression of the *hamp2.7* gene continued to be downregulated, as well as the expression of *hamp2.4* and *hamp2.6*. In addition, the gene expression of *hamp2.7* continued to be downregulated. Besides, the gene expression of *ftha* was downregulated at 3 h p.i. with λ-carrageenin in comparison with fish of the same group sampled at 1.5 h p.i. Furthermore, gene expression of *hamp2.3*, *hamp2.5*, and *hamp2.6* genes was significantly decreased at 6 h p.i. in the HK of fish injected with λ-carrageenin, while the gene expression of *fthb* was not detected. However, the gene expression of *hamp2.4* and *hamp2.7* was upregulated in the HK of fish injected with λ-carrageenin and sampled at 6 h p.i. compared to the fish sampled at 3 h p.i. The expression of *hamp2.1*, *hamp2.2*, *hamp2.8-hamp2.14*, and *tf* genes was not detected in the HK (Fig. 2A).

Regarding the results obtained in the liver, the gene expression of *hamp1* and *ftha* was significantly decreased (*p* < 0.05) in fish injected with λ-carrageenin and sampled at 1.5 and 3 h p.i., respectively, in comparison with the control group (Fig. 2B). In addition, the expression of *hamp2.5*, *hamp2.6*, and *slc40a* genes was also significantly decreased, but only in fish injected with λ-carrageenin and sampled at 6 h p.i. As regards the time factor, the gene expression of *hamp2.3* was downregulated in the liver of fish injected with λ-carrageenin and sampled at 6 h p.i. compared to the fish sampled at 3 h p.i. No significant variations were found in the level of expression in the liver of fish injected with λ-carrageenin of the rest of the genes studied, compared to the values obtained for control fish.

In the case of the skin, no significant alterations (*p* > 0.05) were observed in the expression of any of the genes studied at 1.5 h p.i. in fish injected with λ-carrageenin, compared to control fish. However, the expression of *hamp1* and *hamp2.1* genes was upregulated in fish injected with λ-carrageenin and sampled at 3 h p.i. compared to the expression measured in fish of the control group (Fig. 2C). Furthermore, the expression of *hamp1*, *hamp2.3*, and *hamp2.4* genes was statistically significantly increased in the skin of fish injected with λ-carrageenin and sampled at 6 h p.i. compared to the control group (*p* < 0.05). In the case of the *hamp1* gene, this increase was also significant compared to fish from the same group (injected with λ-carrageenin) and sampled at 1.5 h p.i. In contrast, no significant expression variations were observed neither in the other hepcidin genes studied nor in the genes involved in iron metabolism.

**Fig. 3:**
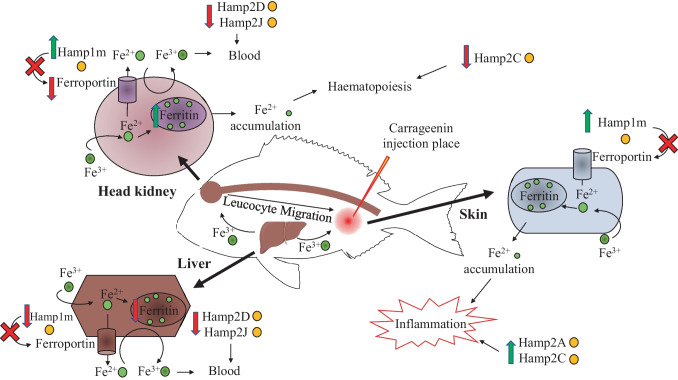
Proposed schematic model of the function of hepcidins in the head, kidney, liver, and skin of gilthead seabream after injection of λ-carrageenin in the in vivo assay. Green and red arrows represent the up- and downregulation of the studied genes, respectively

All the results of the possible mechanism of action of λ-carrageenin relating inflammation and iron metabolism are represented in Fig. 3.

### In Silico Analysis

In silico analysis evidenced that the hepcidin gene *hamp2.1* to *hamp2.14* encoded the mature peptide Hamp2A to Hamp2I, except the following: (i) *hamp2.3* and *hamp2.4* encoded the mature peptide Hamp2C, (ii) *hamp2.5* encoded the peptide Hamp2J, (iii) *hamp2.7*, *hamp2.9*, *hamp2.11*, and *hamp2.13* encoded the peptide Hamp2E, and (iv) *hamp1* encoded Hamp1m (see Table 2).

**Table 2:** Physicochemical and antimicrobial activity of hepcidin mature peptides coded *hamp* genes from gilthead seabream

**Gene**	**Mature peptide**	**Length (aa)**	**Net charge**	R**atio of hydrophobic residues (%)**	**GRAVY**	**W-W hydrophobicity**	**Boman index (kcal mol**^**−1**^**)**	**AMP Probability**^**a**^
*hamp1*	**Hamp1m**	26	+3.25	46	−0.05	−1.58	1.25	0.97
*hamp2.1*	**Hamp2A**	24	−1	54	0.64	0.44	0.56	0.56
*hamp2.2*	**Hamp2B**	20	+1	55	0.50	−0.09	1.41	0.84
*hamp2.3/4*	**Hamp2C**	20	+5	60	0.32	−0.45	2.46	0.88
*hamp2.6*	**Hamp2D**	22	+5	59	0.51	0.44	1.62	0.98
*hamp2.7/9/11/13*	**Hamp2E**	20	+2	60	0.83	−1.88	0.16	0.91
*hamp2.8*	**Hamp2F**	21	0	52	0.31	1.04	1.11	0.62
*hamp2.10*	**Hamp2G**	24	+3	54	0.46	−0.7	0.68	0.97
*hamp2.12*	**Hamp2H**	24	+3	54	0.48	−0.07	0.71	0.97
*hamp2.14*	**Hamp2I**	24	−2	54	0.54	1.94	0.71	0.52
*hamp2.5*	**Hamp2J**	23	+6.25	57	−0.07	−1.14	2.48	0.92

*GRAVY*, grand average hydropathy value of the peptide. *W-W hydrophobicity*, Wimley-White whole-residue hydrophobicity of the peptide. *Boman index*, protein-binding potential

^a^Results with Random Forest from CAMPR3

#### Hepcidin Sequence Analysis

**Fig. 4:**
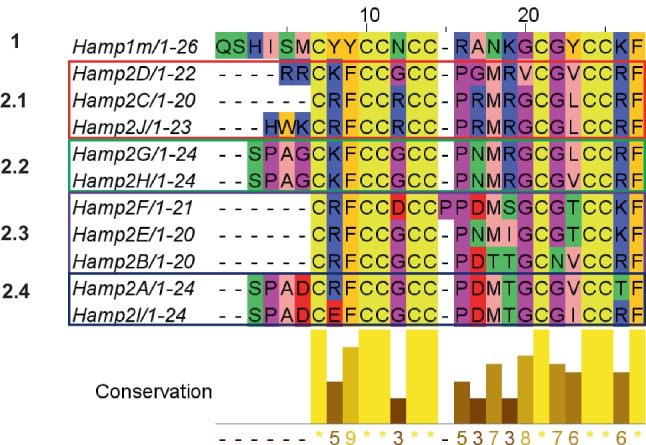
Multiple sequence alignment of mature peptide sequences of gilthead seabream hepcidin proteins. Residues are shown by physicochemical properties following Zappo colors. Below, an amino acid conservation histogram (Jalview) is shown. The colored boxes denote the different groups of hepcidins according to the differences between proteins

The N-t regions of the encoded mature peptides were highly variable among themselves and were organized into 5 clusters of sequences. However, the conservation histogram showed a fully conserved core region with 8 cysteines (marked in yellow in Fig. 4), and 2 glycines (marked in pink in Fig. 4) that was conserved in most of the sequences studied. Hamp1m showed a unique QSHISM motif in the N-t region and 6-tyrosine (Y) instead of phenylalanine (F) as in Hamp2 peptides. Cluster 2.1 was characterized by a high number of positively charged (+) amino acids, especially arginine (R). Hamp2C did not present a clear N-t motif, while Hamp2J and Hamp2D presented a cationic N-terminal motif as HWK and RR, respectively. Hamp2G and Hamp2H displayed a SPAG N-t motif (cluster 2.2), while Hamp2A and Hamp2I displayed a SPAD N-t motif (cluster 2.4). However, although the N-t motifs of Hamp2G and Hamp2H did not add any charge, the SPAD motif added a negatively charged glutamic acid residue (E) to cluster 2.4. Similar to the sequence of Hamp2C, cluster 2.3 did not display any N-t motif. This cluster 2.3 showed three peptides composed of highly polar alcoholic amino acids (S, N, T), as well as two positive amino acids (R/K). In addition, aspartic acid (D) was present in the sequence of Hamp2F/I.

### Hepcidin Physicochemical Analysis

The amino acid sequence of 20 to 26 aa of the eleven mature hepcidin peptides found in gilthead seabream was similar (see Table 2). However, Hamp1m, Hamp2B, Hamp2C, Hamp2D, Hamp2E, Hamp2G, Hamp2H, and Hamp2J showed a positive charge (+3.25, +1, +5, +5, +2, +3, +3, and +6.25, respectively), while the mature peptide of Hamp2A and Hamp2I showed a negative charge (−1 and −2, respectively).

Regarding the hydrophobicity of the hepcidin mature peptides, Hamp1E showed the highest proportion of hydrophobic residues together with Hamp2C (60%), the highest GRAVY value (0.83) and the lowest W-W hydrophobicity value (−1.88) and Boman index value (0.68). In contrast, Hamp1m showed the lowest proportion of hydrophobic residues (46%), Hamp1I the highest W-W hydrophobicity value (1.94), and Hamp1J the lowest GRAVY value (− 0.07), but the highest Boman index (2.48). While Hamp2A, Hamp2F, and Hamp2I have a probability around 50–60% of being AMPs, the rest of the studied peptides have a probability higher of 80% of being AMPs.

#### Enrichment of Hepcidin Promoter Prediction

**Fig. 5:**
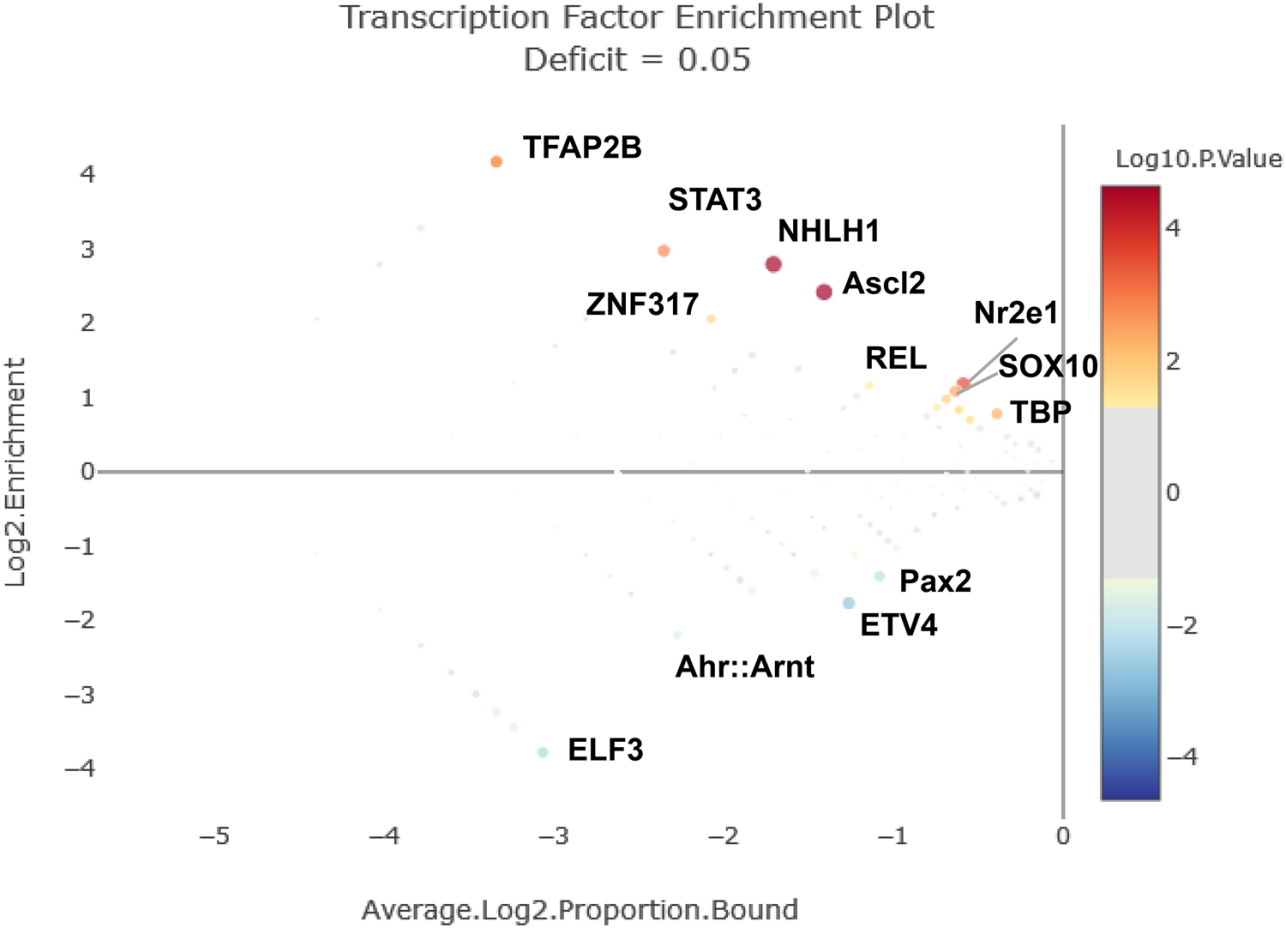
Transcription factor enrichment plot of hepcidin promoters with 21 inflammation-related genes as background. Size and color show ±log10 (*p*-value) (significance score); greater than zero if TF is overrepresented and less than zero if underrepresented

**Fig. 6:**
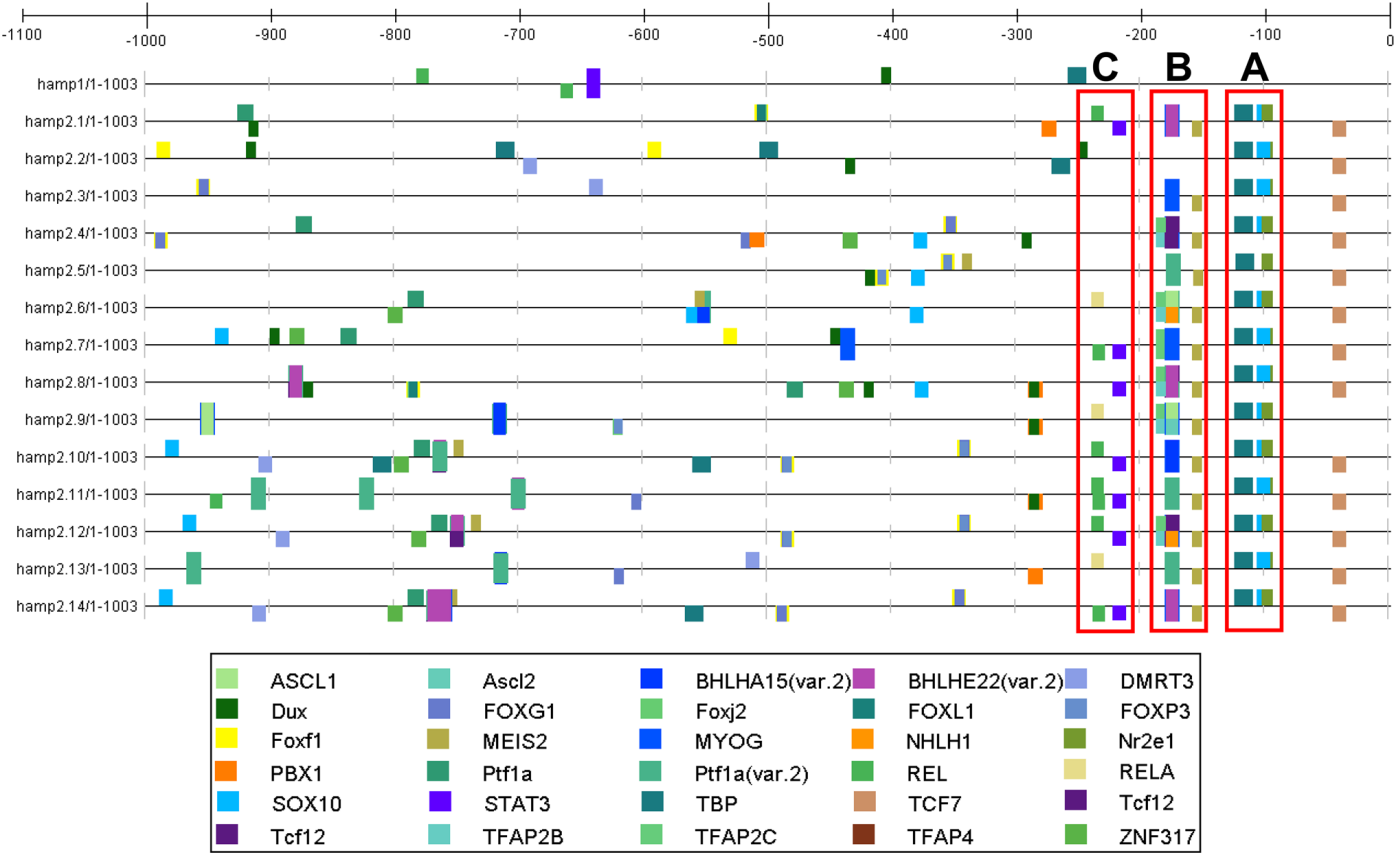
Distribution of 22 overrepresented transcription factor binding sites in 5′-flanking promoter regions of gilthead seabream hepcidin genes (−1000 bp from the start codon). Each color bar indicates a TFBS (above bar, + sense; below bar, − sense); transcription factor types are shown in the lower box. Promoter regions are scaled above the graphical results. This representation of TFBS was constructed with CiiiDER. (For interpretation of references to color in the legend of this figure, the reader is referred to the web version of this article)

Computational enrichment of hepcidin promoters with 21 inflammatory-related genes as background (Table 3) indicated the presence of up to 32 TFBS with *p* ≤ 0.05 (Fisher’s exact test). Of these TFBS, 22 were overrepresented and 10 were underrepresented (Table 4). The most significative and enriched overrepresented TFBS were Ascl2, NHLH1, Nr2e1, STAT3, TFAP2B, SOX10, TBP, ZNF317, and REL (Fig. 5), which mapped to *hamp* promoters (Fig. 6). In contrast, the most significative and enriched underrepresented TFBS were ETV4, ELF3, Pax2, and Ahr:Arnt, which, due to their absence at hepcidin promoters, were not mapped. Briefly, these nine TFBS showed a totally different distribution between *hamp1* and *hamp2* genes, as some TFBS were more upstream (Nr2e1, STAT3, TBP, REL), while others were not identified (Ascl2, NHLH1, TFAP2B, SOX10, ZNF317). Indeed, these TFBS appeared to be clustered in three regions of the *hamp2* promoters: Nr2e1, SOX10, and TBP (cluster A, around −100); Ascl2, NHLH1, TFAP2B, and ZNF317 (cluster B, around −200); STAT3 at −214; and REL at −231 (cluster C). However, the presence of these nine TFBS was variable in some *hamp2* promoters: Ascl2 was in all *hamp2* genes except the *hamp2.2* promoter; NHLH1 was in all hepcidins except *hamp2.2/5*; Nr2e1 and TBP were also in all hepcidin genes; STAT3 was in *hamp2.1/7/8/10/11/12/14*; TFAP2B was in *hamp2.4/6/7/8/9/12*; SOX10 was only in *hamp2* promoters; ZNF317 was in *hamp2.4/6/7/8/10/12/14*; and REL was in *hamp2.1/6/7/9/10/11/12/13/14* (Fig. 6).

**Table 3:** Inflammatory-related genes used to compare TFBS with hepcidin promotors

	**Inflammatory gene name**	**Abbreviation**
**Cell markers**	Colony-stimulating factor receptor 1 receptor	*csfr1*
	Major histocompatibility complex class IIa	*mhcii*
	NADPH oxidase, subunit Phox22	*phox22*
	NADPH oxidase, subunit Phox40	*phox40*
**Cytokines**	Interleukin 1 beta	*il1b*
	Tumor necrosis factor alpha	*tnfa*
	Interleukin-6	*il6*
	Interleukin-7	*il7*
	Interleukin-8	*il8*
	Interleukin-10	*il10*
	Transforming growth factor 1 beta	*tgfβ*
**NF-κB molecules**	v-rel avian reticuloendotheliosis viral oncogene homolog A	*rela*
	v-rel avian reticuloendotheliosis viral oncogene homolog B	*relb*
	v-rel avian reticuloendotheliosis viral oncogene homolog	*rel*
	Nuclear factor of kappa light polypeptide gene enhancer in B-cells 1	*nfkb1*
	Nuclear factor of kappa light polypeptide gene enhancer in B-cells 2	*nfkb2*
**Other inflammatory-related molecules**	Janus kinase 2	*jk2*
	Signal transducer and activator of transcription 3	*stat3*
	Caspase 1	*casp1*
	Cyclooxygenase 2	*cox2*
	Prostaglandin D synthase 1	*pgds1*

**Table 4:** Enrichment analysis of 15 *hamp* genes compared to inflammatory genes detailed in Table 3

**Transcription factor ID**	**Transcription factor name**	**Average Log2 proportion bound**	**Log2 enrichment**	**Significance score**
MA0816.1	Ascl2	− 1.41	2.42	4.65
MA0048.2	NHLH1	− 1.71	2.79	4.59
MA0676.1	Nr2e1	− 0.59	1.18	3.59
MA0144.2	STAT3	− 2.35	2.97	2.83
MA0070.1	PBX1	− 3.34	4.17	2.59
MA0524.2	TFAP2C	− 3.34	4.17	2.59
MA0811.1	TFAP2B	− 3.34	4.17	2.59
MA0442.2	SOX10	− 0.64	1.08	2.41
MA0108.2	TBP	− 0.39	0.78	2.34
MA0521.1	Tcf12	− 0.69	0.98	1.85
MA1593.1	ZNF317	− 2.08	2.06	1.72
MA0774.1	MEIS2	− 0.62	0.83	1.48
MA1100.2	ASCL1	− 0.62	0.83	1.48
MA0614.1	Foxj2	− 0.62	0.83	1.48
MA0850.1	FOXP3	− 0.62	0.83	1.48
MA0613.1	FOXG1	− 0.62	0.83	1.48
MA1619.1	Ptf1a(var.2)	− 0.55	0.70	1.40
MA0500.2	MYOG	− 0.55	0.70	1.40
MA1472.1	BHLHA15(var.2)	− 0.55	0.70	1.40
MA1635.1	BHLHE22(var.2)	− 0.55	0.70	1.40
MA0611.1	Dux	− 0.74	0.87	1.39
MA0101.1	REL	− 1.14	1.16	1.39
MA0092.1	Hand1::Tcf3	− 1.23	− 1.11	− 1.31
MA0711.1	OTX1	− 0.98	− 1.02	− 1.39
MA1534.1	NR1I3	− 1.46	− 1.36	− 1.39
MA0604.1	Atf1	− 1.83	− 1.60	− 1.40
MA0684.2	RUNX3	− 3.34	− 3.23	− 1.52
MA0598.3	EHF	− 3.24	− 3.43	− 1.56
MA0006.1	Ahr:Arnt	− 2.27	− 2.19	− 1.61
MA0067.1	Pax2	− 1.08	− 1.40	− 2.22
MA0640.2	ELF3	− 3.07	− 3.78	− 2.34
MA0764.2	ETV4	− 1.26	− 1.76	− 2.73

## Discussion

In the present study, we used the carrageenin-induced inflammation model in gilthead seabream previously developed by our research group (Campos-Sánchez et al. 2021b) to evaluate the inflammatory function of the multiple copies of hepcidins generated during the vertebrate evolution in seabream (Serna-duque et al. 2022b). Several conditions such as the doses of λ-carrageenin (0 and 1,000 µg mL^−1^) as well as the incubation time (3, 6, 12, and 24 h) for the in vitro assay were selected considering a previous study from our research group to provide a first approximation to hepcidin gene expression in HK (Campos-Sánchez et al. 2022c). However, as homeostatic mechanism seemed to be initiated in in vivo assays at 12 and 24 h after carrageenin injection in seabream, we focused on earlier sampling times (1.5, 3, and 6 h p.i.) (Campos-Sánchez et al. 2021a, b, c, d, 2022a, b). Therefore, different and consistent expression patterns of hepcidins were found in HK (particularly in leucocytes), liver, and skin during the inflammatory response caused by intramuscular injection of λ-carrageenin. Furthermore, to clarify the different functions of the hepcidins studied, an in silico analysis of hepcidin sequences, as well as physicochemical and antimicrobial activities, was developed, pointing out remarkable differences that will be listed and discussed below.

Previous studies in gilthead seabream have shown that hepcidins are highly expressed in acidophilic granulocytes (AGs) compared to monocytes/macrophages and lymphocytes (Cuesta et al. 2008). AGs are able to exert their functions through the release of hydrolytic enzymes such as myeloperoxidase, lysozyme, and other proteases contained in their cytoplasmic granules (Kolaczkowska and Kubes 2013; Campos-Sánchez et al. 2021a). Therefore, hepcidins could also be stored in AG granules and released after being induced. As for our in vitro assay, we followed the scheme shown in Fig. 1B to facilitate the understanding of the results obtained. Thus, gene expression of *hamp1*, *ftha*, and *fthb* was upregulated in HKLs at 3, 6, and 12 h of incubation with λ-carrageenin. However, the *slc40a* gene was downregulated under the same conditions.These data suggest that λ-carrageenin could induce iron oxidation (from Fe^3+^ to Fe^2+^) in HKLs (mainly in AGs) and its sequestration in the form of ferritin, as well as inhibition of iron-exporting ferroportin by Hamp1m, favoring iron storage (Schmidt 2015). In this regard, we hypothesize that the stored iron could be utilized in hematopoiesis (Caldas et al. 2016) following the release of hepcidin plasmids, Hamp2A (*hamp2.1*) and Hamp2C (*hamp2.3*), in order to provide new leucocytes ready to be activated upon recognition of an external agent such as λ-carrageenin. Furthermore, the hepcidins Hamp2E, Hamp2F, Hamp2G, and Hamp2H (*hamp2.9* to *hamp2.13*), whose probability of being AMPs was greater than 90%, were expressed only in HK but not in the liver or skin of fish injected with λ-carrageenin in the in vivo assay. Therefore, their function seems to be more related to their possible antimicrobial activity than to their involvement in inflammatory response or iron metabolism (Katzenback 2015).

To try to understand the gene expression results of the in vivo assay in the three organs (HK, liver, and skin) studied in the present work, we will follow the scheme in Fig. 3. First, we evaluated the gene expression of hepcidins in the HK, which is the main hematopoietic organ (functionally analogous to mammalian bone marrow) of seabream (Meseguer et al. 1995; Calder et al. 2013). Thus, upregulation of *hamp1*, *fthb*, and *slc40a* gene expression in the HK of fish injected with λ-carrageenin and sampled at 1.5 h p.i. could be associated with the promotion of iron storage inside leucocytes, removing it from plasma (hypoferremia). Thus, iron would not be available as a nutrient for invading bacteria, avoiding exacerbation of the inflammatory response already produced. Furthermore, this iron could be utilized in the normal process of hematopoiesis that takes place in HK, as previously discussed. In addition, activated leucocytes (in response to the presence of λ-carrageenin) could migrate from the HK to the inflamed area to develop their specific functions (Kolaczkowska and Kubes 2013; Campos-Sánchez et al. 2021a). Interestingly, previous studies developed by our research group pointed to AGs (leucocytes with high expression of hepcidin genes) as the main cells involved in the inflammatory response triggered by λ-carrageenin, being these cells, the first ones recruited from the HK to the inflamed zone (Campos-Sánchez et al. 2021d). Furthermore, we have determined that our model of acute inflammation is characterized by reaching the highest peak of response at 3 h p.i. and initiating the regulation and termination of inflammation at 6 h p.i. (Campos-Sánchez et al. 2021b, c, 2022b). This fact could be related to the downregulation in HK of hepcidins *hamp2.4* and *hamp2.6*, and *hamp2.3*, *hamp2.5*, and *hamp2.6* at 3 and 6 h p.i., respectively. Interestingly, elevated levels of hepcidin expression have been linked to increased iron storage in RBC recycling macrophages and hepatocytes, and to limited iron absorption from dietary sources (Schmidt 2015). Conversely, decreasing hepcidin expression levels may facilitate the release of nonheme iron from liver and macrophage stores, increasing iron transfer across intestinal epithelial cells to effectively control the supply of bioavailable iron (Schmidt 2015). In this sense, we hypothesize that cytokines induced in leukocytes could result in an increase in hepcidin expression, decreasing the number of hematopoietic progenitors to match the decrease in the amount of iron available for hematopoiesis, once inflammation is under control. Therefore, hepcidins could play a key role in this regulatory process by exhibiting a cyclic tendency.

On the other hand, the liver is known to be one of the main organs involved in iron homeostasis, as well as the main source of hepcidins (and other AMPs) and inflammatory proteins (Schmidt 2015). In the present study, the downregulation of *hamp1* and *ftha* gene expression at 1.5 and 3 h p.i., respectively, could be related with the mobilization of iron from the liver to other organs such as the HK or skin, where it could play a crucial role. However, further studies are needed to prove this thesis. Moreover, both Hamp2D (*hamp2.6*) and Hamp2J (*hamp2.5*) showed the highest Boman index, so they seem to have a high potential to bind to other proteins, probably mediating in the process of iron mobilization from the liver to other organs. In this sense, the downregulation of the *slc40a*, *hamp2.5*, and *hamp2.6* genes could point to the activation of regulatory mechanisms to reduce iron release, as discussed above. This fact is also consistent with the onset of the termination of inflammation produced by λ-carrageenin at 6 h p.i. (Campos-Sánchez et al. 2021c, 2022b).

Finally, the skin, which constitutes the first active mechanical, physical, chemical, and biological barrier in fish (Bullock and Roberts 1975; Elliott 2000), was also selected in the present work to evaluate the effects of λ-carrageenin injection. Notably, it has been described that in response to various stimuli, specific and nonspecific effector cells are able to migrate through the circulatory system into the skin, triggering an inflammatory response (Bos 1977; Parkhurst and Saltzman 1994; Cumberbatch et al. 2003). In fact, we have shown by immunohistochemistry that in the skin of seabream specimens, a rapid increase in cutaneous mucus-secreting cells, as well as in the number of AGs, was observed in the vicinity of the λ-carrageenin injection site (Campos-Sánchez et al. 2021d). Consistent with those results, in the present study, the upregulation of the *hamp1* gene in the skin of fish sampled at 3 and 6 h p.i. of λ-carrageenin could be related to the recruitment of leucocytes (mainly AGs) from the HK to the skin. Once in the skin, the AGs could start releasing cytokines, hydrolytic enzymes, hepcidins, and other HDPs to resolve the triggered inflammatory process (Kolaczkowska and Kubes 2013; Campos-Sánchez et al. 2021a). In fact, it has been observed that proinflammatory cytokines such as IL-6 or bone morphogenic protein (BMP) rapidly increase hepcidin expression (Costa et al. 2011; Steinbicker et al. 2011). This fact could associate the increased expression of hamp2.1 genes at 3 h and *hamp2.3* and *hamp2.4* in the skin of fish injected with λ-carrageenin and studied at 6 h p.i. with some proinflammatory functions. In other words, these results suggest that Hamp2A and Hamp2C could be acute-phase proteins involved in the inflammatory response, in agreement with the functions described for hepcidins in mammals (Gerwick et al. 2007; Lin et al. 2007). These results are also in agreement with an in vivo study developed in European sea bass (*D. labrax*) challenged with *Vibrio anguillarum* and injected with a synthetic hepcidin (hep20). Briefly, the expression of several cytokines (IL-1β, TNFα, and IL-10) was increased 1, 3, and 7 days after hep20 injection. However, the expression of the cytokines studied was reduced at 14 and 21 days compared to control fish, highlighting first their inflammatory function and then their regulatory role during inflammation produced by pathogenic infection (Álvarez et al. 2022). On the other hand, since hepcidins seem to be regulated only at the transcriptional level (Nicolas et al. 2002; Adamsky et al. 2004), the overrepresented presence of various inflammatory TFBS such as STAT3 or NF-κB (REL and RELA) located in their promoter regions could be a very relevant data in the nexus of induction and regulation of inflammation by hepcidins in seabream. However, further studies would be necessary to determine the function of these TFBS and their actual involvement in the inflammatory process.

In conclusion, gene expression and bioinformatics analyses developed in the present study seem to indicate that iron metabolism and inflammation are closely related, being the multiple copy of hepcidins found in gilthead seabream one of the possible cornerstones connecting both processes in this fish species. In this sense, the HK, the liver, and the skin seem to show an integrated response contributing to the initiation and development of inflammation produced by λ-carrageenin. The present results provide new approaches in the study of inflammation in fish of commercial interest, providing a basis for future studies.

## Data Availability

The data are available on the DIGITUM institutional repository from the University of Murcia: http://hdl.handle.net/10201/130524 (accessed 1 May 2023).
